# Double trouble! Concomitant distal ulna fractures predict worse 1-year outcome in distal radius fractures: a registry-based cohort study of 5,536 patients

**DOI:** 10.2340/17453674.2025.44352

**Published:** 2025-08-15

**Authors:** Linnea ARVIDSSON, Marcus LANDGREN, Anna Kajsa HARDING, Antonio ABRAMO, Magnus TÄGIL

**Affiliations:** 1 Department of Clinical Sciences Lund, Orthopaedics, Skåne University Hospital and Lund University, Lund, Sweden;; 2 Department of Orthopaedic Surgery, Hand Surgery Unit, Copenhagen University Hospital, Herlev and Gentofte, Gentofte, Denmark; 3 Department of Hand Surgery, Skåne University Hospital, Malmö, Sweden

## Abstract

**Background and purpose:**

Data on distal radius fractures (DRFs) with concomitant metaphyseal distal ulna fractures is limited. We aimed to determine whether a combined DRF and distal ulna fracture (DRUF) predicts a worse patient-reported outcome, measured by the Disabilities of the Arm, Shoulder, and Hand (DASH) score, 1 year after injury.

**Methods:**

This prospective registry-based cohort study included 5,536 adult patients with a DRF between 2003 and 2018. The 1-year DASH scores were recorded. All DRUFs were identified. Multivariable binary logistic regression assessed whether the presence of a distal ulna fracture predicted a 1-year DASH score > 35, indicating severe upper-extremity symptoms.

**Results:**

259 of 5,536 patients (4.7%) had a DRUF. Their mean age was 73 years (SD 15), and 86% were women. The median 1-year DASH score was higher in the combined fracture group compared with those with a DRF only (23, interquartile range [IQR] 5–45] vs 9, IQR 2–27, P < 0.001). A DRUF increased the odds of a 1-year DASH > 35 by 97% (OR 1.97, 95% confidence interval [CI] 1.40–2.75, P < 0.001). Surgical fixation of the DRF in DRUF patients was associated with lower odds of a worse outcome (OR 0.44, CI 0.23–0.85, P = 0.02). Distal ulna fracture fixation did not affect 1-year DASH (P = 0.7).

**Conclusion:**

The odds of having a DASH > 35, indicating severe symptoms, almost doubled at 1 year in patients with a DRUF compared with those with a DRF only.

A distal radius fracture (DRF) is often accompanied by an ulnar styloid fracture [1,2]. A concomitant metaphyseal fracture of the distal ulna is less common, occurring in only 2–9% of DRFs [3-5], and there is limited evidence regarding patients with combined distal radius and distal metaphyseal ulna fractures, here termed DRUFs [2].

The distal ulnar metaphysis is important for DRF stability. However, distal ulna fracture management is rarely discussed, and optimal treatment remains unclear [2,6-8]. In the few published studies of DRUFs, most authors recommend surgical fixation of the distal ulna in young and active patients if the fracture remains unstable after DRF fixation [2,9]. Yet, most patients with DRUFs are elderly with poor bone quality [2]. When DRUFs are treated surgically, standard techniques are used for fixation of the DRF. However, osteosynthesis of the distal ulna fracture, particularly in osteoporotic bone, can be technically challenging, and no consensus exists regarding the preferred ulnar fixation method [2,10,11]. In an elderly patient with severe osteoporosis, resection of a highly comminuted ulnar head is an alternative [2,8]. Comparable clinical and radiological results have been reported in elderly patients following both surgical and non-surgical treatment [8,12-15]. However, all series are small, and most often highlight the knowledge gap.

Radiographic healing is a limited indicator of successful treatment. Even with fracture union, the patient’s functional level may decline, and larger studies incorporating patient-reported outcomes are needed [16].

Our primary aim was to investigate whether a DRUF was a predictor of a worse 1-year DASH score compared with a DRF only.

## Methods

### Design

This was a single-site, registry-based cohort study assessing whether a combined DRF and distal ulna fracture, i.e., the DRUF, affects patient-reported outcomes, as measured by DASH, 1 year after injury. The study is reported according to STROBE guidelines.

### The Lund Distal Radius Fracture Registry

Since 2003, all patients aged 18 years and older presenting with a metaphyseal DRF to the emergency department at Skåne University Hospital in Lund have been prospectively and consecutively registered in the Lund Distal Radius Fracture Registry. One secretary has reviewed the medical records of the emergency department weekly to identify cases of wrist fractures. All patients aged 18 years or older with a DRF are included, with no exclusion criteria applied. Patients were included in the registry based on the ICD-10 codes S52.50/51 and S52.60/61. Data collection continued through December 31, 2018, after which the registry was phased out due to the implementation of a national fracture registry. The present study was designed and conducted retrospectively using data from the registry.

According to local clinical guidelines at the time [17,18], non-displaced DRFs were treated with a forearm cast for 4–5 weeks. Displaced DRFs were reduced and immobilized in a cast. If reduction failed or secondary displacement occurred by the time of the 10-day follow-up, surgical treatment was recommended. The proportion of surgical vs non-surgical treatment of the DRFs remained constant during the years 2003–2012 [17]. There were no guidelines regarding treatment of a concomitant distal metaphyseal ulnar fracture and these were treated according to the surgeon’s preference, either conservatively or using closed pinning of the ulna fracture or open surgery using wire forms or ulnar plates.

### Outcome measures

The primary outcome was the DASH score, a patient-reported outcome measure, assessing disabilities and symptoms related to the upper extremity. Scores range from 0 to 100, with higher scores indicating higher disabilities.

As part of the registry protocol, the DASH questionnaire was sent to all patients 12 months after their injury. Non-responders received a reminder 2 weeks later. In 2008, the original 30-item DASH was replaced by the shorter 11-item QuickDASH to improve response rates. The 2 versions are strongly correlated (Spearman’s ρ = 0.97, P < 0.001) [18]. Both versions have been validated in Swedish [19,20]. When comparing QuickDASH and DASH scores, QuickDASH tended to show slightly higher values. This difference was statistically significant, but not considered clinically relevant [19]. As the 2 scores are generally regarded as interchangeable [19], the term “DASH” will be used throughout this article, despite the data including both versions.

### Study participants and variables

All 5,536 patients in the registry were included. To avoid the impact of coding errors and to ensure differentiation between ulnar styloid and distal metaphyseal ulnar fractures, the emergency department radiographs were retrospectively reviewed by an orthopedic surgeon. Patients with a DRF and a concomitant metaphyseal distal ulna fracture were classified as DRUFs. Those with a DRF, with or without an ulnar styloid fracture, were classified as DRF-only.

Age, sex, fracture type, and DASH were recorded in the registry. Additional variables, such as comorbidity, polypharmacy, fracture classification, open vs closed fracture, and type of treatment, were retrieved through medical record review. Due to resource constraints, this manual review was performed only for the DRUF patients and an equally sized, randomly selected, age- and sex-matched subset of DRF-only patients. Matching was performed to reduce imbalance, as the age and sex distribution of the DRUF group differed from that of the full cohort. For each patient with a DRUF, a patient with an isolated DRF matched by sex and age at time of injury was randomly selected. In the youngest and oldest age groups, the number of patients was limited and sex was prioritized over age, and an age difference of less than 3 years was accepted. No additional exclusion criteria were applied.

Comorbidity was assessed using the Charlson Comorbidity Index (CCI), which scores chronic conditions using weights of 1, 2, 3, or 6 based on severity and mortality risk [21]. For analysis, the CCI was dichotomized into < 2 vs ≥ 2. Age, although part of the original CCI, was not included in the score in this study, as it was analyzed separately. Polypharmacy was defined as 5 or more prescribed medications.

**Figure 1:**
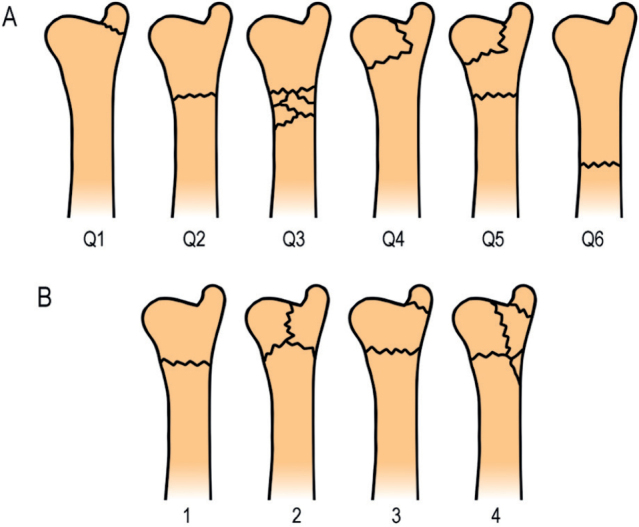
Classification of distal ulna fractures. AO Q-modifier subclassification, Q1–Q6 (Q1 = ulnar styloid base fracture, Q2 = simple neck fracture, Q3 = comminuted neck fracture, Q4 = ulnar head fracture, Q5 = ulnar head and neck fracture, and Q6 = fracture of ulnar diaphysis). @@@Biyani classification system for ulnar metaphyseal fractures in the setting of DRFs (1 = simple extraarticular fracture with minimal comminution, 2 = inverted T- or Y-shaped fracture with an ulnar styloid fragment including a portion of the metaphysis, 3 = fractures of the lower end of the ulna with avulsion fracture of the ulnar styloid, and 4 = comminuted fractures of the lower ulnar metaphysis, with or without styloid fracture).

All DRUFs were classified by an orthopedic surgeon using the AO Q-modifier and the Biyani classification for distal ulna fractures [3,22] (classification systems are illustrated in Figure 1). To assess intra- and interobserver reliability, 10% of DRUF radiographs were independently reviewed twice (6 months apart) by a hand surgery specialist and an orthopedic resident. Interobserver agreement was substantial for both the Biyani classification (κ = 0.78) and the AO Müller classification (κ = 0.67).

### Sample size

No power analysis was performed prior to the study. All cases from the registry were included in the main analyses.

### Statistics

DASH is an ordinal variable with a skewed distribution and was dichotomized into > 35 vs ≤ 35. A 1-year DASH > 35 has previously been associated with major disability [17]. A multivariable binary logistic regression model was used to assess the association between DRUF status and a DASH > 35, adjusting for age and sex. To assess the robustness of the chosen cut-off, a post-hoc sensitivity analysis was performed using alternative DASH thresholds (25, 30, 40, and 45), and findings were consistent (see Supplementary Table 1).

A second logistic regression was performed on the smaller dataset (DRUF patients and matched DRF-only patients), adjusting for age, sex, CCI score, polypharmacy, open vs closed fracture, treatment type (surgical or non-surgical), and AO classification. Variables with a P value < 0.25 in univariable analysis were included in the multivariable model.

DASH scores were reported as median and interquartile range (IQR), and group comparisons were made using the Mann–Whitney U test. Normally distributed continuous variables were reported as mean and standard deviation (SD), with group differences analyzed using a 2-sample t-test. Categorical variables were presented as counts and percentages, and differences in distribution were tested using the chi-square test. Interobserver agreement for fracture classification was assessed using Cohen’s kappa.

Trends in the proportion of surgically treated DRFs (2003–2018) were assessed using linear regression analysis, both for all DRFs and separately for DRUFs.

To describe the uncertainty of each estimate, 95% confidence intervals (CIs) are presented alongside P values. A 2-sided P value < 0.05 was considered statistically significant. Data was analyzed using SPSS version 29 (IBM Corp, Armonk, NY, USA).

### Ethics, data sharing plan, funding, and disclosures

The study adhered to the principles of the Declaration of Helsinki. Approval was obtained from the Regional Ethical Review Board in Lund, which waived the need for informed consent (ETIK 2009/318). Data is available upon reasonable request. The study was supported by the Swedish Government Grant for Clinical Research (ALF), Region Skåne Research Funds, and the Maggie Stephens, Erik and Angelica Sparre, Greta and Johan Kock, and Alfred Österlund Foundations. The authors report no conflict of interests. Complete disclosure of interest forms according to ICMJE are available on the article page, doi: 10.2340/17453674.2025.44352

## Results

### Patients

**Figure 2:**
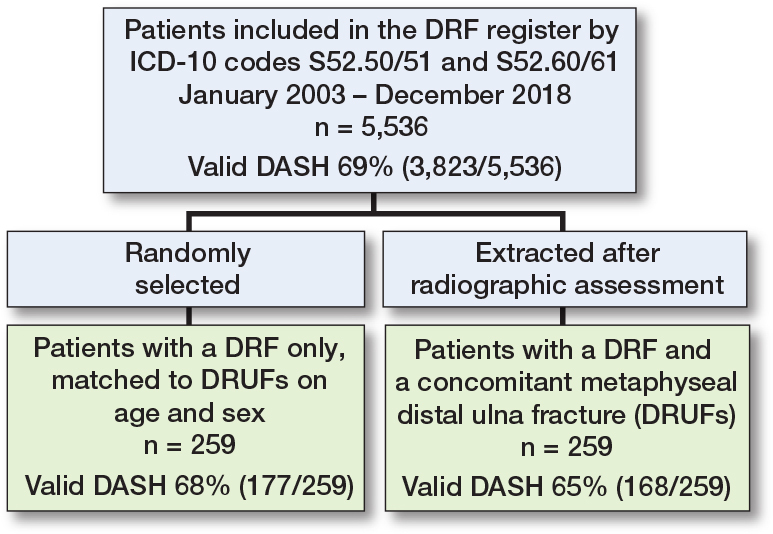
Flowchart of patients included in the study.

Between January 2003 and December 2018, 5,536 patients were included in the register. Among these, 259 patients (4.7%) had a concomitant distal ulna fracture (a flowchart of the inclusion process is shown in Figure 2).

The overall response rate for the DASH questionnaires in the registry was 69% (3,823/5,536) and for the DRUF subgroup 65% (169/259). Baseline patient characteristics are summarized in Table 1. For the secondary analysis including additional covariates, characteristics of the matched subset of DRF-only patients are listed in Supplementary Table 2.

**Table 1:** Baseline characteristics for all 5.536 DRF and DRUF patients. Values are count (%) unless otherwise specified

Item	DRF	DRUF	P value
No. of patients	5,277 (	259 (	
Male	1,176 (22)	37 (14)	< 0.001
Female	4,101 (78)	222 (86)	< 0.001
Mean age (SD)	62 (18)	73 (15)	< 0.001
CCI > 2		94 (36)^**a**^	
Polypharmacy		75 (29)^**b**^	

DRF = distal radius fracture; DRUF = distal radius and distal metaphyseal ulna fracture; CCI = Charlson Comorbidity Index.

a Missing data for 2 patients.

b Missing data for 15 patients.

### Fracture classifications and treatment

There was no statistically significant linear trend in the proportion of DRFs (P = 0.09) or DRUFs (P = 0.4) treated surgically over time. Fracture classifications for DRUFs are set out in Table 2. Details of surgical and non-surgical treatment for DRUFs are provided in Table 3.

**Table 2:** Fracture classifications of DRUF patients (n = 259)

Item	n (%)
DRF AO	
AO – A	127 (49)
AO – B	4 (1.5)
AO – C	128 (49)
Distal ulna fracture Biyani	
Biyani 1	82 (32)
Biyani 2	60 (23)
Biyani 3	62 (24)
Biyani 4	55 (21)
Distal ulna fracture AO Q-modifier	
AO-Q 1	N/A
AO-Q 2	88 (34)
AO-Q 3	13 (5.0)
AO-Q 4	29 (11)
AO-Q 5	127 (49)
AO-Q 6	2 (0.4)
Open fractures	41 (16)

For abbreviations, see Table 1 and AO-Q = the classification for ulnar styloid fractures,

N/A = not applicable.

**Table 3:** Treatment of DRUF patients (n = 259)

Surgically	111
Fixation of DRF	111
Volar plate	73
Fragment-specific fixation	18
External fixation	13
Combination/other method	7
Fixation of distal ulna fracture	49
Ulnar sledge	11
Ulnar pin plate	14
Other ulnar plate	11
Cerclage	7
K-wire	1
Combination	5
Non-surgically	148
Weeks in cast^**a**^	
1–5 weeks	123
> 5 weeks	11
Type of cast^**b**^	
Non-circulated	123
Below elbow	136

For abbreviations, see Table 1.

a Missing data for 14 patients.

b Missing data for 2 patients.

### Patient-reported outcome

**Figure 3:**
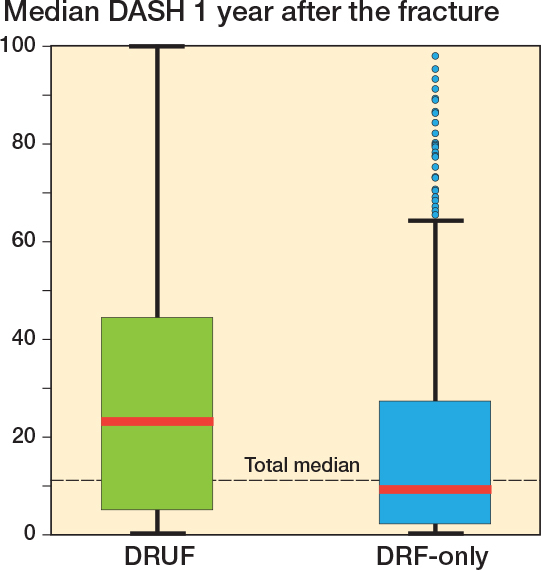
Box plot of median DASH 1 year after fracture, comparing median DASH for patients with a distal radius and distal metaphyseal ulna fracture (DRUF) with patients who have only a distal radius fracture (DRF).

In the DRUF group, the DASH score at 1 year was inferior to the DRF-only group (23 [IQR 5–45] vs 9 [IQR 2–27]; P < 0.001) (Figure 3). A greater proportion of patients with DRUFs reported a 1-year DASH > 35: 36% vs 18% in the DRF-only group (P = 0.01).

In the multivariable logistic regression model, the presence of a DRUF was associated with a 97% increase in the odds of having a DASH > 35 at 1 year (OR 1.97, CI 1.40–2.75, P < 0.001) (Table 4).

**Table 4:** Univariable and multivariable binary logistic regression analyses assessing the association between predictor variables and a 1-year DASH of > 35 in 5,536 patients

Predictor of 1-year DASH > 35	OR (CI)	P value
Univariable analyses		
Age (continuous)	1.03 (1.03–1.04)	< 0.001
Sex (female vs male)	1.90 (1.50–2.41)	< 0.001
Fracture (DRUF vs DRF-only)	2.56 (1.84–3.55)	< 0.001
Multivariable analyses		
Age (continuous)	1.03 (1.03–1.04)	< 0.001
Sex (female vs male)	1.42 (1.11–1.82)	0.005
Fracture (DRUF vs DRF-only)	1.97 (1.40–2.75)	< 0.001

CI = 95% confidence interval; DASH = Disabilities of the Arm and Shoulder and Hand; DRF = distal radius fracture; DRUF = distal radius and distal ulnar metaphyseal fracture; OR = odds ratio.

In the secondary regression analysis (with DRUF patients and the matched DRF-only subset) several additional covariates were included (CCI, polypharmacy, fracture type, surgical vs non-surgical treatment, open vs closed fracture, and AO classification). Univariable analyses for these variables showed that each additional year of age increased the odds of a poor DASH outcome by 5% (OR 1.05, CI 1.03–1.07, P < 0.001). Patients with DRUFs had higher odds of a 1-year DASH score > 35 (OR 1.82, CI 1.14–2.90, P = 0.03), as did patients with open fractures (OR 2.^13^, CI 1.03–4.41, P = 0.04). Comorbidity (CCI ≥ 2) (OR 2.87, CI 1.78–4.62, P < 0.001) and polypharmacy (OR 4.60, CI 2.77–7.77, P < 0.001) were the strongest predictors of poor outcome. Sex and AO classification of the DRF were not associated with the 1-year DASH outcome (Supplementary Table 3). After adjustment, having a DRUF remained a significant predictor of a worse outcome (DASH > 35) at 1 year (OR 2.35, CI 1.33–4.16, P = 0.003) (Supplementary Table 4).

A secondary finding in this model was that surgical treatment of the DRF (with or without fixation of the distal ulna fracture) was associated with significantly lower odds of a worse outcome (OR 0.44, CI 0.23–0.85, P = 0.02). Addition of distal ulna fixation alongside DRF fixation did not affect the median 1-year DASH score in the DRUF group (15 [IQR 0–42] after DRF fixation only vs 19 [IQR 6–35] for both DRF and DUF fixation; P = 0.7).

Surgically treated DRUF patients were younger (mean age 67 years, SD 14) than those treated non-surgically (mean age 78 years, SD 15; P < 0.001) and had fewer comorbidities (mean CCI 1.0, SD 1.4 vs 1.6, SD 1.6; P = 0.004).

## Discussion

We aimed to determine whether a DRUF predicts a worse patient-reported outcome, measured by the Disabilities of the Arm, Shoulder, and Hand (DASH) score, 1 year after injury. We found that patients with a DRUF had significantly higher odds of major disability at 1 year than patients with a DRF only. This association remained significant even after adjusting for age, sex, comorbidity, polypharmacy, and fracture characteristics. Although DRUFs are relatively uncommon (5% in our cohort), this finding highlights their potential clinical impact.

In line with previous studies, our results show that DRUFs primarily affect older women [2,4] and most often are associated with AO type A or C DRFs, but rarely with type B fractures [4]. Both the AO-Q and Biyani classification systems demonstrated only substantial interobserver reliability, which aligns with previous studies [23]. Notably, the rate of open fractures among DRUFs was unexpectedly high at 16%.

Sweden’s national guidelines for DRFs emphasize that there is a lack of evidence-based treatment recommendations regarding concomitant distal ulna fractures [24]. It is up to each treating physician to determine the appropriate method for further treatment. In our cohort, both surgical techniques and nonoperative management strategies (including type and duration of immobilization) differed widely, reflecting the absence of standardized treatment protocols in the department.

Our data showed that surgically treated DRUF patients had better outcomes than those treated non-surgically. While this may partly be explained by confounding factors—such as the surgically treated group being younger and having fewer comorbidities—surgery remained significantly associated with better outcomes even after adjustment.

While our results suggest that fixation of the distal ulna fracture may not significantly influence the 1-year DASH outcome, these findings should be interpreted cautiously. The sample size was limited, with only 34 patients undergoing surgical fixation of the distal ulna fracture and completing the DASH questionnaire.

Given the rarity of DRUFs, a registry-based randomized controlled trial comparing surgical and non-surgical treatment could be a feasible approach for future research.

### Strengths

A major strength of this study is the use of a large prospective registry, which enabled us to analyze an uncommon but potentially important fracture combination.

### Limitations

Although patients were registered and DASH collected prospectively, additional clinical variables, including comorbidities and treatment details, were collected retrospectively from the medical records, which may have affected data quality and contributed to missing values.

Nonetheless, the primary regression analysis was performed using a complete dataset for the independent variables.

DASH measures function and symptoms across the entire upper extremity. Other conditions affecting the shoulder, elbow, and hand may influence the DASH independently of the wrist fracture. Moreover, while the DASH response rate was relatively high (69%), we did not perform a non-responder analysis for this study. However, a previous analysis of a subset of this register cohort found that non-response was more common among patients younger than 40 or older than 80 years, men, and those treated nonoperatively [17].

Due to resource constraints, not all covariates were available for the entire cohort. To address this, we conducted a secondary regression analysis using all DRUF patients and a subset of age- and sex-matched DRF-only patients. Analyzing only this subset introduces a loss of power and potential selection bias, and analyzing the full cohort would be preferable. However, the similar effect estimates in both analyses support the assumption that there is a true correlation.

Although this study includes a relatively large number of cases, it was conducted at a single center, which may limit the external validity of the findings. As the patient group consists almost exclusively of elderly patients, the findings may not be transferable to a younger patient population.

### Conclusion

The odds of having a DASH > 35, indicating severe symptoms, almost doubled at 1 year in patients with a DRUF compared with those with a DRF only.

*In perspective,* the finding that combined distal radius and distal ulna fractures predict poorer outcomes can be valuable knowledge for both the treating physician and the patient, as it may aid in shared decision-making and justify closer follow-up.

There is a need for better treatment strategies to enhance patient outcomes.

### Supplementary data

Supplementary Tables 1–4 are available as supplementary data on the article page, doi: 10.2340/17453674.2025.44352

## Supplementary Material



## Notes

LA: methodology, investigation, formal analysis including statistical analysis, writing—original draft. ML: investigation, data curation, writing—review and editing. AKH: investigation, writing—review and editing. AA: software, writing—review and editing. MT: supervision, project administration, validation, writing—review and editing.

Handling co-editor: Taco Gosens

*Acta* thanks Michel E. H. Boeckstyns and Hebe Désirée Kvernmo for help with peer review of this manuscript.
