# Supplementary material for: Double trouble! Concomitant distal ulna fractures predict worse 1-year outcome in distal radius fractures: a registry-based cohort study of 5,536 patients
Source: Acta Orthop. 2025 Aug 15;96:606–11. doi: 10.2340/17453674.2025.44352 (PMC12357178; doi:10.2340/17453674.2025.44352)
Supplement: Supplementary file 1 [file ActaO-96-44352-s1.pdf]

Supplementary data

Supplementary Table 1. Multivariable binary logistic regression analyses, adjusted for age and sex, assessing the association between having a DRUF and different cut-offs for DASH in 5,536 patients.			
**Predictors**	**OR**	**CI**	**P value**
Predictor of 1-year DASH >25 (DRUF vs. DRF-only)	2.10	1.53–2.91	<0.001
Predictor of 1-year DASH >30 (DRUF vs. DRF-only)	2.32	1.68–3.21	<0.001
Predictor of 1-year DASH >40 (DRUF vs. DRF-only)	2.11	1.49–2.98	<0.001
Predictor of 1-year DASH >45 (DRUF vs. DRF-only)	1.69	1.15–2.48	0.008
CI = 95% confidence interval; DASH = Disabilities of the Arm and Shoulder and Hand, DRF = distal radius Fracture, DRUF = distal radius and distal ulnar metaphyseal fracture: OR = odds ratio.			

Supplementary Table 2. Patient and fracture characteristics of the DRF-controls matched to DRUFs by age and sex (n = 259)	
**AO-classification**	n (%)
A	135 (52)
B	9 (3.5)
C	115 (44)
**Open fracture**	2 (0.9)
**Type of cast**	
Non-circulated	197 (99)
Below elbow	205 (97)
**Time in cast for non-surgically treated (**mean 4.2 [SD 0.7]**)**	
1–5 weeks	181 (99.5)
>5 weeks	1 (0.5)

CCI	
0–1	159 (61)
2–5	95 (37)
≥ 6	5 (1.9)
**Polypharmacy**	69 (28)
**Type of treatment**	
Surgical	52 (20)
Volar plate	38 (73)
Fragment specific	9 (17)
External fixation	5 (10)
Combination/other	0
CCI = Charlson Comorbidity Index; DRF = distal radius fracture; DRUF = distal radius and distal ulnar metaphyseal fracture.	

Supplementary Table 3. Univariable binary logistic regression analyses assessing the association between predictor variables and a 1-year DASH of >35 in 518 patients (DRUFs = 259 and DRF-only = 259)

**Predictor of 1-year DASH >35**	**OR**	**CI**	**P value**
Age (continuous)	1.05	1.03–1.07	<0.001
Sex (female vs. male)	2.31	0.99–5.38	0.053
Fracture (DRUF vs. DRF-only)	1.82	1.14–2.90	0.03
AO-Classification of the DRF B vs. A	0.58	0.12–2.74	0.58
AO-Classification of the DRF C vs. A	1.52	0.95–2.43	0.08
Treatment (surgery vs. no surgery)	0.57	0.35–0.95	0.03
CCI (≥2 vs. <2)	2.87	1.78–4.62	<0.001
Polypharmacy (yes vs. no)	4.60	2.77–7.77	<0.001
Open fracture (yes vs. no)	2.13	1.03–4.41	0.04

CCI = Charlson Comorbidity Index; CI = 95% confidence interval; DASH = Disabilities of the Arm and Shoulder and Hand, DRF = distal radius fracture, DRUF = distal radius and distal ulnar metaphyseal fracture.

Supplementary Table 4. Multivariable binary logistic regression analyses assessing the association between predictor variables and a 1-year DASH of >35 in 518 patients (DRUFs = 259 and DRF-only = 259)

**Predictor of 1-year DASH >35**	**OR**	**CI**	**P value**
Age (continuous)	1.02	0.99–1.04	0.08
Sex (female vs. male)	2.36	0.90–6.21	0.08
Fracture (DRUF vs. DRF-only)	2.35	1.33–4.16	0.003
AO-Classification of the DRF, B vs. A	0.76	0.13–4.31	0.8
AO-Classification of the DRF, C vs. A	1.78	1.03–3.07	0.04
Treatment (surgery vs. no surgery)	0.44	0.23–0.85	0.02
CCI (≥2 vs. <2)	1.17	0.63–2.20	0.6
Polypharmacy (yes vs. no)	3.69	1.98–6.86	<0.001
Open fracture (yes vs. no)	1.60	0.65–3.94	0.3

For abbreviations, see Supplementary Table 3
